# Fetus-in-fetu: A Rare Congenital Anomaly

**DOI:** 10.4103/2006-8808.73621

**Published:** 2010

**Authors:** Saurabh Kumar Gupta, Parul Singhal, Neera Arya

**Affiliations:** *Department of Surgery, Aryan Hospital & Research Center, Lower Bazar, Modinagar - 201 204, Ghaziabad, Uttar Pradesh, India*

**Keywords:** Abdominal lump, axial skeleton, fetus-in-fetu, retroperitoneal, teratoma

## Abstract

Two cases of fetus-in-fetu, on which we performed surgery in 2003 and 2006, are being reported. Both the cases presented with a lump in the abdomen. Radiology confirmed the diagnosis. The lumps were found in the retroperitoneum and successfully excised. Because of the rarity of the condition, these two cases are being reported with relevant salient features and are discussed in the light of available literature.

## INTRODUCTION

**Figure 1:**
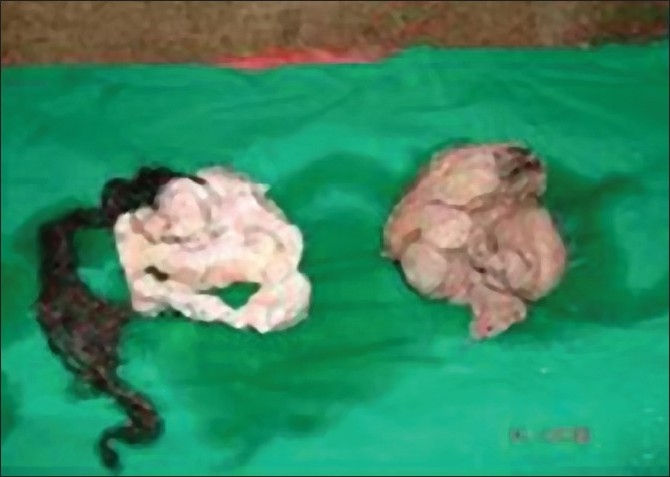
Gross specimen of both fetuses after excision of sac

Fetus-in-fetu (FIF) is a rare entity in which one malformed vertebrate fetus is enclosed within the body of its twin. This is an extremely rare condition, and Hopkins *et al*. found less than 100 case reports on extensive review of the literature. An array of presentations is described in the literature although the embryo-pathogenesis and differentiation from a teratoma have not been well established. Two unique cases are reported here and the literature is reviewed [Figure 1].

## CASE REPORTS

### Case 1

**Figure 2:**
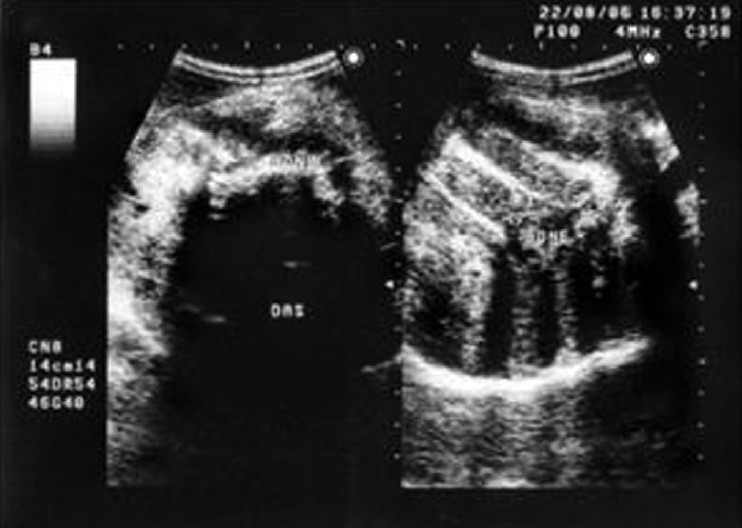
USG showing bony structures with distal acoustic shadows

**Figure 3:**
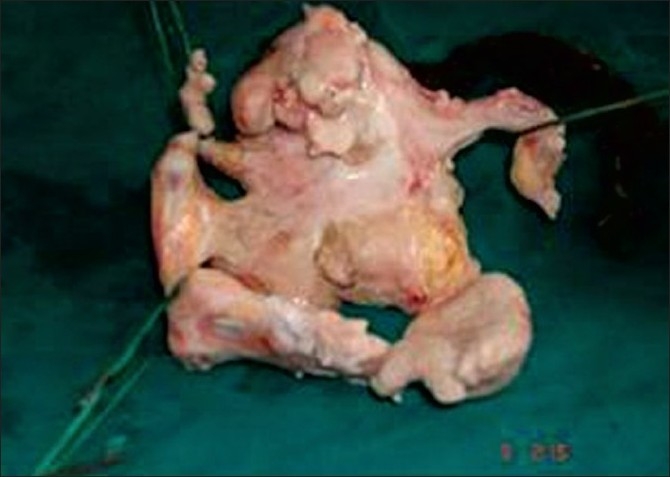
Fetus with well-developed upper and lower limbs with external genitalia

**Figure 4:**
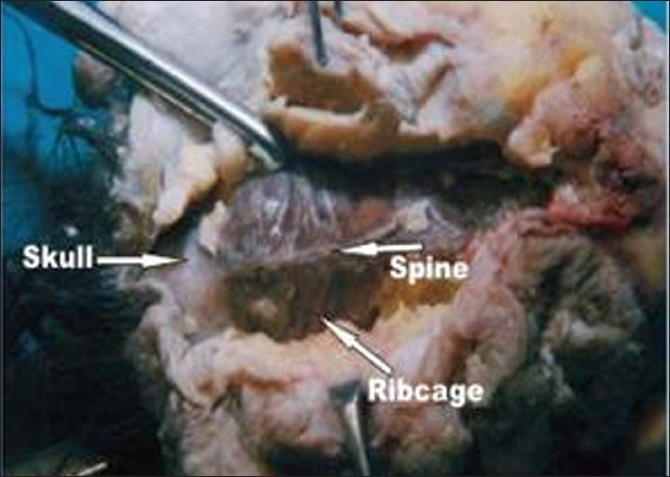
Dorsal dissection showing spine, rib cage, and skull

A young boy of 9 years presented in the outpatient department in 2006 with chief complaints of swelling in the right hypochondrium, with a dull ache of 3 months duration. On general examination, the child was found to be normal except for the presence of slight pallor. On local examination, we found fullness in the right hypochondrium with a round intra-abdominal, non-tender mass of variable consistency. The lump extended into the right lumbar and epigastric regions. Lower margins of the lump extended up to 6 cm below the right coastal margin just crossing the midline. However, upper margins could not be palpated. The lump was firm, and finger insinuation was possible between the lump and right coastal margin. It was not moving with respiration, and a dull note was present all over the mass. There were no abnormal sounds auscultated over the swelling. Plain skiagram of the abdomen revealed a soft tissue mass in the right upper abdomen along with the calcified bony structures. Ultrasonography (USG) of the abdomen showed a solid cum cystic complex mass of 10 × 9 cm with multiple linear and rounded echogenic foci and distal acoustic shadows, suggestive of bony parts seen superior to the right renal region with mild hepatosplenomegaly [Figure 2]. Intravenous pyelography of the patient revealed bilateral normally excreting kidneys with a soft tissue mass displacing the right kidney downward. Computerized tomography (CT) scan of the patient showed a well-defined, rounded, heterogenous retroperitoneal mass of size 10.2×9.7×10.5 cm in the right pararenal space. The mass consisted of areas of fluid and fat attenuation, calcific densities to the shape of long bones and vertebrae, along with some soft tissue attenuating areas. On IV contrast administration, walls of the mass showed mild to moderate contrast enhancement. Fat planes between the mass and the adjacent structures were well maintained. After doing all the routine investigations, the mass was excised successfully by right supraumbilical transverse approach. The peritoneum was opened and there was a large tumor retroperitoneally in the right hypochondrium, densely adherent to the diaphragm superiorly and to the right kidney inferiorly. Large feeding vessels were seen entering into the tumor directly from the aorta. Inferior vena cava (IVC) was stretched and densely adherent to the anterior surface of the tumor. By meticulous dissection, the tumor was separated from the surrounding structures including IVC. The feeding vessels were ligated and divided. The tumor was excised *in toto*. Gross specimen of the tumor measured 12×10×10 cm and weighed 600 g. On incising the membranous amniotic sac, a well-formed fetus was delineated embedded in thick pultaceous material. The fetus was anencephalic, with well-developed trunk, upper and lower limbs and external genitalia (penis and scrotum) [Figure 3]. Surprisingly, the fetus had 30 cm long hair. On further dissection of the fetus from the dorsal side, a well-developed vertebral column was found along with a rib cage [Figure 4]. Skull bone was seen, and a burr hole was made and biopsy taken. On dissecting the abdomen, it was found to be divided into two compartments with no identifiable intra-abdominal structures. Brain biopsy subsequently proved to be of primitive neural tissue.

### Case 2

**Figure 5:**
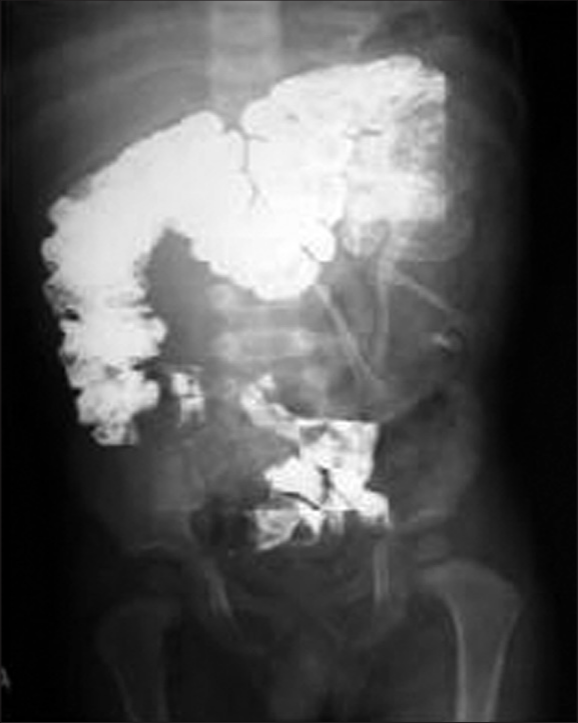
Barium radiology showing displacement of bowel loops and long bones

**Figure 6:**
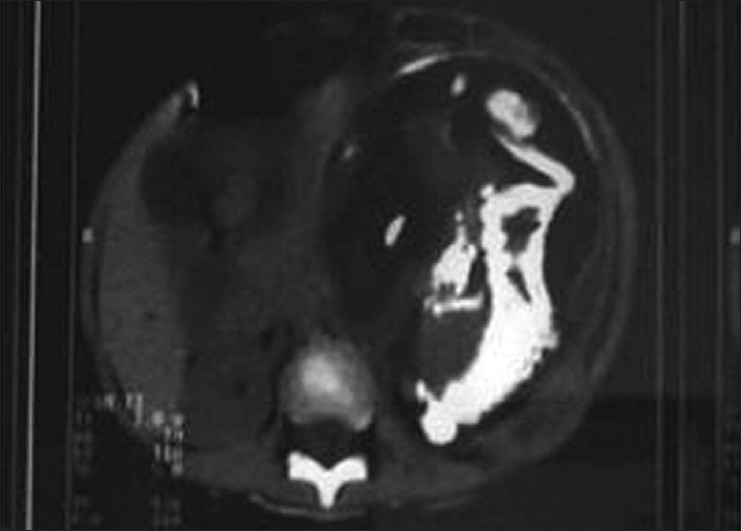
CT scan showing large retroperitoneal mass with a variable consistency

**Figure 7:**
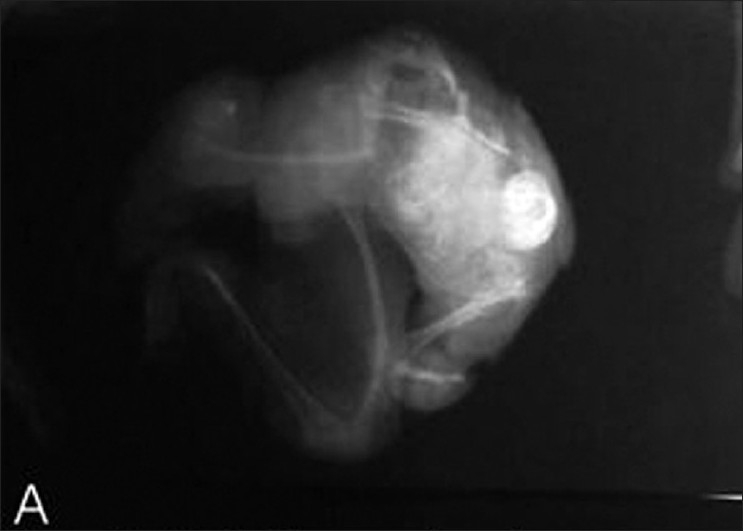
Plain skiagram of specimen showing well-formed bony structures

**Figure 8:**
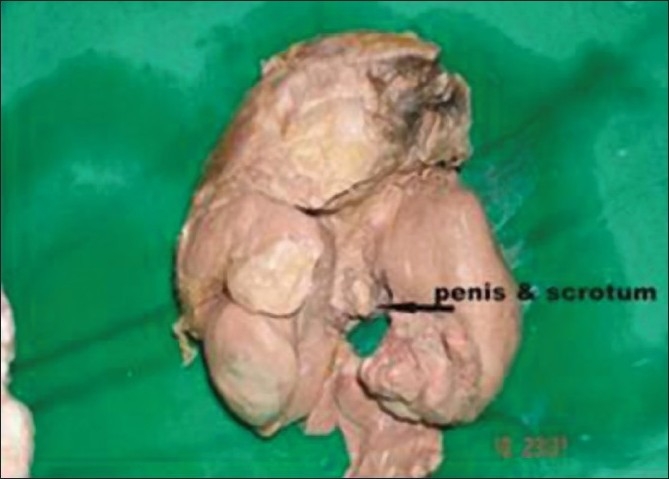
Gross specimen of fetus showing well-differentiated external genitalia

An 18-month-old male child presented in 2003 with gradually increasing abdominal swelling. The mother noticed some fullness of abdomen a few days after the birth of the child. The mother noticed a well-formed abdominal lump at 3 months of age with a gradual increase in swelling. Poverty, ignorance, the precious nature of the child, and fear of surgery all contributed to the delayed presentation. The child was born of a normal pregnancy, and there was no history of twins in the family. On examination, the child was found to be anemic and malnourished. A large abdominal lump was occupying the whole of the left half of the abdomen. The lump was round, non-tender, and of variable consistency. Bones were palpated. Plain X-ray showed a soft tissue mass in the left abdomen with bones and calcification, giving a suspicion of FIF. Barium meal follow-through revealed displacement of small bowel loops on the right side [Figure 5]. Three-dimensional (3D) ultrasonography showed a well-formed spine and arranged skeletal system. CT scan of the patient revealed a large retroperitoneal mass with variable (solid and cystic) consistency, extending from the left hypochondrium to the pelvis and displacing the left kidney superolaterally and posteriorly along with the spleen laterally. The internal structure of the mass showed fluid, fat, soft tissues, and bony elements [Figure 6]. After thorough evaluation and correction of anemia, fluid, and electrolyte imbalances, the patient was subjected to surgery under general anesthesia by a liberal transverse muscle cutting incision in the left abdomen. A mass was found in the retroperitoneum adherent to surrounding structures and major vessels. The mass was enclosed in a complete membranous sac, supplied by major vessels originating from the aorta. It was separated from surrounding structures and excised *in toto*. The abdomen was closed in layers without drainage. Postoperative recovery was normal. The excised specimen measured 10 × 8 × 7 cm and weighed 800 g. Plain skiagram of the specimen showed well-formed bony structures [Figure 7]. Upon opening the sac, a well-formed fetus was seen. It had a rudimentary head (anencephaly) and well-developed thorax, abdomen, upper limbs with fingers, lower limbs with feet and toes, and well-differentiated external genitalia (penis and scrotum) [Figure 8]. Both the patients were followed postoperatively for 6 months and 3 years, respectively, and were doing well with no complaints.

## DISCUSSION

Fetus-in-fetu, a term quoted by Lewis,[1] was first described by Meckel to describe a rare condition in which a malformed vertebrate fetus with organogenesis was found inside the body of its partner, usually in the abdominal cavity. It represents an aberration of diamniotic, monochorionic, monozygotic twinning in which unequal division of the totipotent inner cell mass of the developing blastocyst leads to the inclusion of a smaller cell mass within a maturing sister embryo. However, some researchers believe that FIF represents one spectrum of malignant teratoma.[2] The incidence of FIF is 1 per 500,000 births[3] with fewer than 100 cases reported worldwide. According to Willis, the presence of an axial skeleton distinguishes a teratoma from an FIF.[4] The presence of a separate spinal column indicates that the fetus has passed through a primary stage of gastrulation involving formation of neural tube, metamerization, and symmetrical development around this axis.[5] The diagnosis of FIF is doubtful when the mass does not obviously contain vertebral axis. The most common presentation is an abdominal mass that is typically located in the upper retroperitoneum. However, other locations (cranium, intrahepatic, pelvis, neck)[6–9] have also been described. Symptoms of FIF occur mainly due to its mass effect that includes abdominal distension, emesis, jaundice, a pressure effect on the renal system, and dyspnea. Occasionally, the anomaly is asymptomatic.[10] The preoperative diagnosis of FIF depends on the radiological findings. Plain abdominal X-rays may be helpful in diagnosis, with up to almost half of the cases showing the presence of a vertebral column and axial skeleton.[11] The 3D ultrasonography and CT scan have now further enhanced the accuracy of preoperative diagnosis. In recent years, magnetic resonance imaging (MRI) has also been used to diagnose FIF. Cases of reported FIF weighed between 13 g[12] and 2000 g.[13] The size and weight of the fetus is likely to be related to its blood supply. Fetuses with distinct vascular connections to the host are larger with better developed features. However, both our specimens had well-developed fetoid features with a spinal column and long bones, joints, clavicle, rib cage, scapula, and external genitalia with a well-formed pelvis and head containing neural tissue. In conclusion, FIF is a very rare and interesting entity that typically presents as an abdominal mass in infancy and early childhood. Using current imaging modalities, it can be diagnosed fairly accurately before surgery. Complete excision, which requires meticulous dissection, is curative and allows confirmation of diagnosis.

## References

[1] Lewis RH (1960). Foetus in Foetu and retroperitoneal teratoma. Arch Dis Child.

[2] de Lagausie P, de Napoli Cocci S, Stempfle N, Truong QD, Vuillard E, Ferkadji L (1997). Highly differentiated teratoma and fetus-in-fetu: A single pathology?. J Pediatr Surg.

[3] Grant R, Pearn JH (1969). Foetus-in-foetu. Med J Aust.

[4] Willis RA (1962). The Borderland of Embryology and Pathology.

[5] Gonzalez Crussi F (1982). Extragonadal Teratoma. Atlas of tumor pathology.

[6] Goldstein I, Jakobi P, Groisman G, Itskovitz-Eldor J (1996). Intracranial fetus-in-fetu. Am J Obstet Gynecol.

[7] Magnus KG, Millar AJ, Sinclair-Smith CC, Rode H (1999). Intrahepatic Fetus-in-fetu: a case report and review of literature. J Pediatr Surg.

[8] Chua JH, Chui CH, Sai Prasad TR, Jabcobsen AS, Meenakshi A, Hwang WS (2005). Fetus-in fetu in the pelvis: report of a case and literature review. Ann Acad Med Singapore.

[9] Borges E, Lim-Dunham JE, Vade A (2005). fetus in fetu appearing as a prenatal neck mass. J Ultrasound Med.

[10] Hopkins KL, Dickson PK, Ball TI, Ricketts RR, O’Shea PA, Abramowsky CR (1997). Fetus-in-fetu with malignant recurrence. J Pediatr Surg.

[11] Hoeffel CC, Nguyen KQ, Phan TT, Truong NH, Nguyen TS, Tran TT (2000). Fetus-in-fetu: a case report and literature review. Pediatrics.

[12] Lee EY (1965). Foetus in Foetu. Arch Dis Child.

[13] Pantankar T, Fatterpekar GM, Prasad S, Maniyar A, Mukherji SK (2000). Fetus in fetu: CT appearance - report of two cases. Radiology.

